# Conceptual Design and Electromagnetic-Thermal Coupling Analysis of Superconducting Current-Limiting Reactor Under Self-Triggered Built-In Magnetic Field Excitation

**DOI:** 10.3390/ma19163419

**Published:** 2026-08-12

**Authors:** Qinghe Yu, Hao Xu, Xiaoyuan Chen, Bo Wang, Han Zhao, Junfei Yang, Qiang Xu, Ke Qing

**Affiliations:** 1 School of Engineering, Sichuan Normal University, Chengdu 610101, China; 2023180165@stu.sicnu.edu.cn (Q.Y.); 20242195016@stu.sicnu.edu.cn (H.X.); 20242195012@stu.sicnu.edu.cn (B.W.); 20252181029@stu.sicnu.edu.cn (H.Z.); 20252181019@stu.sicnu.edu.cn (J.Y.); xuqiang@sicnu.edu.cn (Q.X.); 2 Sichuan Energy Internet Research Institute, Tsinghua University, Chengdu 610213, China

**Keywords:** superconducting current-limiting reactor, superconducting magnet design, resistive-type superconducting fault-current limiter, self-triggered quenching

## Abstract

Conventional resistive-type superconducting fault-current limiters (RSFCLs) rely exclusively on fault currents and temperature increases to trigger quenching, resulting in delayed fault response and excessive heat buildup under short-circuit conditions. To mitigate these limitations, this paper proposes a self-triggered, magnetic-field-excited superconducting current-limiting reactor (SCLR) integrated with a solenoidal magnet assembly. During the design and simulation phases, a segmented discretization method is employed to quantitatively characterize the gradient distribution of the external perpendicular field within the superconducting tapes and coils. This approach theoretically elucidates the mechanism by which spatially non-uniform magnetic fields influence current-limiting performance. DC short-circuit simulations show that the background magnetic field instantly reduces the critical current, rapidly transitioning the superconducting layer into a nonlinear resistive state. In contrast to the conventional topology, the proposed SCLR achieves two key performance improvements during short-circuit faults: it limits the peak fault current to just 45.9% of the value recorded with the conventional RSFCL scheme, and it reduces the maximum temperature rise by 1.4 K. The findings of this study provide a theoretical foundation and technical references for multi-field coupling modeling and structural optimization of magnetic-field-regulated current-limiting devices in DC grids.

## 1. Introduction

With the large-scale grid integration and operation of DC distribution networks and renewable energy converter systems, the amplitude of short-circuit fault current continues to rise, placing stringent requirements on the response speed and thermal stability of fault-current limiting and protection devices [1]. Conventional protective equipment such as series reactors and fuses suffer from high power loss and poor adaptability, and they lack automatic recovery function, making them incapable of realizing fast protection [2]. Resistive-type superconducting fault-current limiters (RSFCLs) rely on the high impedance naturally generated during a superconductor’s quenching to suppress fault currents without additional external triggering control [3,4]. Featuring fast response and automatic recovery to a superconducting state after fault clearance, an RSFCL holds broad engineering potential in DC transmission and distribution scenarios [5,6,7,8].

Traditional RSFCL devices rely solely on fault-induced Joule heating to reduce critical currents, resulting in an obvious thermal hysteresis effect during current limiting operation. The limiting impedance builds up slowly at the initial stage of fault occurrence, and the huge short-circuit impulse current may easily lead to thermal burnout of superconducting tapes and serious damage to primary grid equipment [9,10]. Meanwhile, most existing studies have adopted the E-J power law to treat SFCLs as ideal variable resistors [11,12], overlooking the real-time temperature variation during current limiting operations. While a small number of studies incorporate temperature variables [13,14,15], the electromagnetic-thermal coupling modeling of RSFCLs adopts the simplifying assumption of a uniform background magnetic field, neglecting the gradient effect of the magnetic field distributed along the extended arc length of superconducting coils. Accordingly, such models fail to accurately characterize the spatially differentiated evolution laws of critical currents, dynamic quenching resistance of each segmented coil unit, and current sharing behaviors of multiple coating layers during the fault transient process [16,17,18,19,20].

In order to simultaneously address the issues of slow quenching speed and long recovery time of traditional SFCL devices, this paper proposes a novel racetrack-type RSFCL topology integrated with a solenoid-biased background magnetic field. In the proposed scheme, the racetrack current-limiting coil is embedded inside a solenoidal magnet. The perpendicular field continuously generated by the magnet pre-degrades the intrinsic critical current. Furthermore, a fully coupled electromagnetic-thermal time-domain simulation framework embedded with a segmental field-dependent critical current model is established, which quantitatively evaluates the transient electrical and thermal responses induced by non-uniform perpendicular field. The developed framework can reproduce the key features of the magnetic-field-assisted current-limiting process, and it provides a feasible numerical tool for structural-parameter optimization and multi-physics coupling characteristic analysis.

## 2. Structural Design of the Superconducting Current-Limiting Reactor

**Figure 1:**
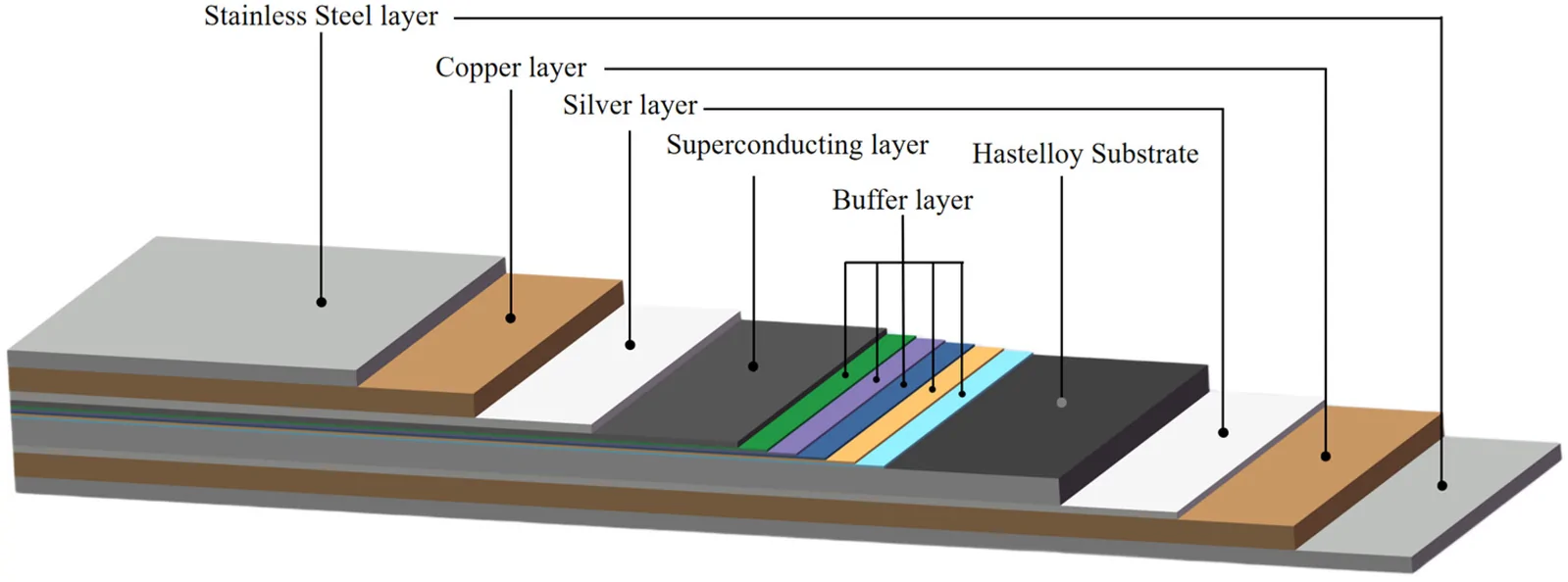
Schematic diagram of the multi-layer structure inside a REBCO coated tape.

The commercial REBCO tapes from Shanghai Superconductor Technology Co., Ltd. are adopted as the conductors of the current-limiting non-inductive coil in this work. The tape features a multi-layer stacked composite architecture, comprising a Hastelloy substrate, buffer stack, REBCO superconducting layer, silver stabilizer layer, copper layer and stainless steel coating in sequence from the interior to the exterior [21,22]. A schematic cross-section of the tape is shown in Figure 1.

The Hastelloy substrate delivers a superior mechanical performance, greatly boosting the structural robustness and thermal stability of the superconducting tape under elevated temperatures [23,24,25]. Stainless steel layers are pre-packaged on both sides of the tape. Joule heat generated by the coil during quenching can be rapidly transferred to the 77-K liquid nitrogen medium through the stainless steel layers. The silver, copper and stainless steel layers form parallel shunting branches. During the quenching period, a fault current can be diverted to the metal layers to avoid thermal burnout of the superconducting layer caused by bearing the excessive current alone [26,27,28].

Previous studies have compared the electromagnetic characteristics of REBCO double racetrack coils (DRC) and double pancake coils (DPC) under alternating background magnetic fields [29]. The straight segments of racetrack coils are exposed to stronger perpendicular magnetic field components, and this matches our design principle of utilizing a bias magnetic field to realize the current limitation. Moreover, this topology facilitates electromagnetic analysis and numerical modeling [30,31,32], supporting the characteristic simulation of the RSFCL unit.

Stacked double-pancake coil assemblies are widely used to construct superconducting background magnets [33,34]. References [35,36] proposed a superconducting air-core DC reactor for voltage smoothing and fault-current-limiting applications, and they presented numerical calculation methods for the inductance and critical current characteristics of solenoidal magnets.

On this basis, a modular stacked double-pancake scheme is selected for the external background solenoidal magnet. Composed of six series-connected double-pancake coil assemblies, the magnet features axial gaps of roughly 20 mm, 18 mm, 16 mm, 18 mm and 20 mm between adjacent assemblies from top to bottom [35]. This variable gap structure can effectively adjust the magnetic field direction at the edges of both sides of the magnet, significantly reducing the perpendicular magnetic field acting on the winding coils in the edge region, thereby increasing the critical current of the entire magnet and avoiding potential quenching risks.

**Figure 2:**
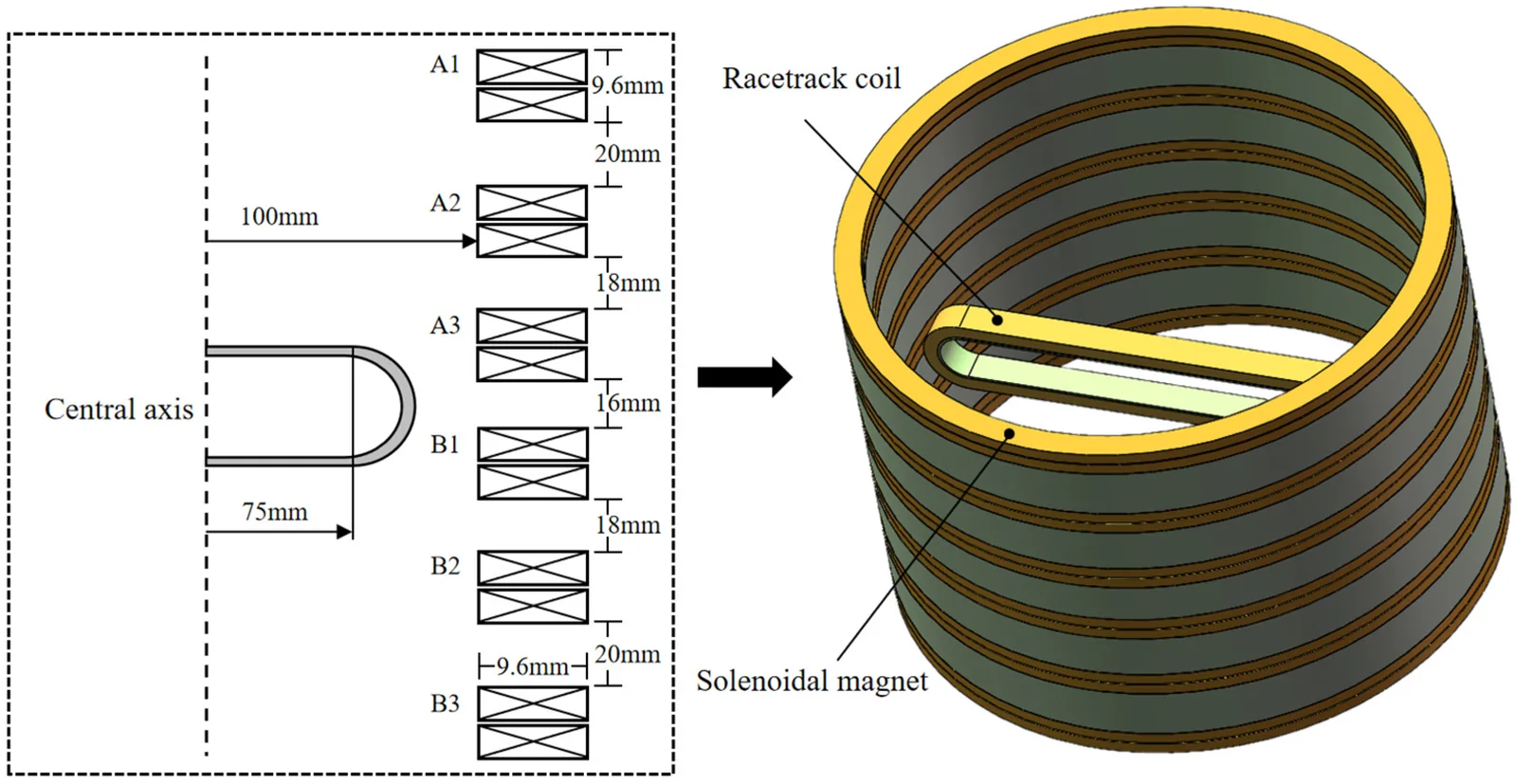
Overall structural schematic representation of the superconducting current-limiting reactor.

A racetrack non-inductive coil is employed as the current-limiting winding and placed inside the external solenoidal magnet. The perpendicular magnetic field produced by the solenoid penetrates the plane of the racetrack coil to build a stable background bias field. The three-dimensional configuration of the whole device is shown in Figure 2, and Table 1 and Table 2 summarize the geometric parameters of the racetrack coil and the solenoidal magnet, respectively.

## 3. Electromagnetic-Thermal Coupled Model

(1) Temperature-Dependent Resistance Model for Metal Layers

The resistivities of silver, copper and stainless steel layers inside the superconducting tapes are temperature-dependent. A temperature coefficient is introduced to characterize the resistance variations induced by temperature rise. The unified resistance *R*_x_ for a single conductor layer is written as [14]:

(1) $$R_{x}\;=\frac{\;(\rho_{x}\;+\;(T\;-77)\;\times \;K_{x})\;\times \;l_{t}}{S_{x}},$$

where *ρ*_x_ denotes the benchmark resistivity of metal at 77 K, taken as 0.27 × 10^−8^ Ω∙m, 0.19 × 10^−8^ Ω∙m and 56.8 × 10^−8^ Ω∙m for silver, copper and stainless steel, respectively; *K*_x_ represents the resistivity temperature coefficient of silver, copper and stainless steel; *S*_x_ is the cross-sectional area of each layer; and *l*_t_ is the total tape length used for the racetrack-type coil.

In this model, the silver, copper and stainless steel substrates are assumed to have ideal longitudinal electrical contacts. Therefore, the interfacial contact resistance and current transfer length among different metallic layers are not explicitly considered, and the three metallic layers are treated as an equivalent parallel shunt path [11,14]. The total normal-conducting resistance can be calculated by:

(2) $$R_{\mathrm{normal}}\;={\;R}_{\mathrm{Ag}}∥R_{\mathrm{Cu}}∥R_{\mathrm{SS}}.$$

(2) Nonlinear Power-Law Model for Superconducting Layer

A universal E-J power law is adopted to describe the voltage-current characteristic of superconductors [19,37]. The expressions for the instantaneous voltage *U*_s_ and resistance *R*_s_ of the superconducting layer are written as:

(3) $$U_{s}\;=\;l_{t}\;\times \;E_{c}\;\times \;{\left(\frac{I_{s}}{I_{c}}\right)}^{n}+L\frac{di}{dt}\;\mathrm{and}$$

(4) $$R_{s}=\frac{U_{s}}{I_{s}},$$

where *E*_c_ is the critical electric field of the superconductor, taken as 0.1 mV/m; *n* is the power-law index, taken as 25; and *L* is the inductance of superconducting series smoothing inductor, taken as 10 mH.

The critical current *I*_c_ of the superconductor varies with the temperature and magnetic field, which is formulated as [19,38,39]:

(5) $$I_{c0}\;=\;\frac{I_{c}\left(T_{0}\right)}{{\left(1\;-\frac{T_{0}}{T_{c}}\right)}^{\beta }}\;\mathrm{and}$$

(6) $$I_{c}(T,B_{z})=I_{c0}{\left(1-\frac{T}{T_{c}}\right)}^{\beta }{\left(1+\frac{B_{z}}{B_{1}}\right)}^{-\alpha },$$

where *I*_c0_ is the initial critical current at 77 K under zero magnetic field; *T*_c_ is the critical temperature of the superconductor; *β* is the temperature degradation index; *B*_z_ is the perpendicular component of the magnetic flux density; and *α* and *B*_1_ are anisotropy factors, taken as 0.65 and 0.049 T, respectively [36].

(3) Transient Temperature Rising Model Based on Thermal Balance

Joule heat generated by the superconducting layer and metal shunting layers acts as the heat source of the system during fault quenching. The real-time thermal energy is transferred to the 77 K liquid nitrogen medium via convection through stainless steel layers. A lumped-parameter heat balance equation is adopted to calculate temperature variation in time domain.

The net heat generation within one simulation time step is expressed as [30]:

(7) $$\Delta Q\;=\int_{t_{1}}^{t_{2}}(U_{\mathrm{total}}I_{\mathrm{total}}-P_{\mathrm{cool}})dt,$$

where the cooling power *P*_cool_ removed via liquid nitrogen can be calculated by [19]:

(8) $$\;P_{\mathrm{cool}}\;=\;h\;\times \;A\;\times \;\Delta T^{\prime },$$

where *A* stands for the heat transfer surface area, which is determined by the actual conductor length and winding configuration. Δ*T*′ is the temperature difference between the superconducting tape and liquid nitrogen.

The equation for calculating the convective heat transfer coefficient *h* of stainless-steel is as follows [14,40]:

(9) $$h\;=\;649.928\;-\;303.82\;\times \;\Delta T^{\prime }+\;52.35\;\times \;\Delta T^{\prime 2}-\;1.565\;\times \;\Delta T^{\prime 3}.$$

Then, the temperature rise of the whole conductor is expressed as [14]:

(10) $$\Delta T\;=\frac{\Delta Q}{C_{v}\times m}.$$

The fitted function of specific heat capacity for stainless steel over the temperature range of 50–300 K can be expressed as [14,40]:

(11) $$C_{v}=3.1\times 10^{-5}\times T^{\;3}-0.022\times T^{\;2}+5.81\times T-138.3.$$

Temperature is updated iteratively at each time step, and the temperature at the next time step equals the temperature at the previous time step plus the inter-step temperature increment [19]:

(12) $$T_{n+1}\;={\;T}_{n}\;+\;\Delta T.$$

(4) Equivalent Modeling of the Segmented Perpendicular Background Field

**Figure 3:**
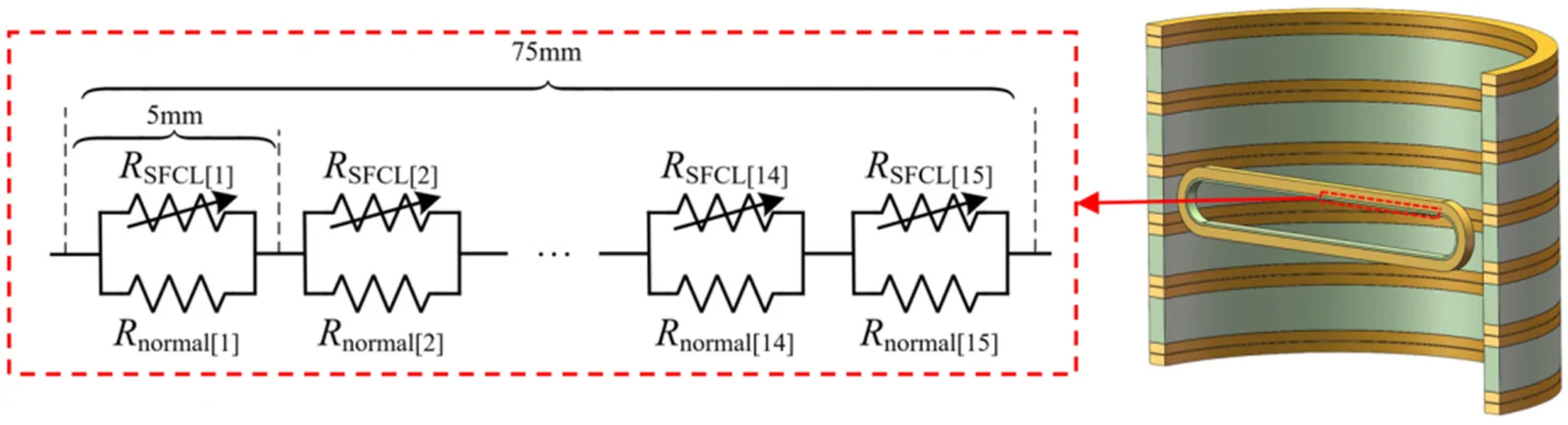
Equivalent circuit of the 15-segment RSFCL model.

Conventional RSFCL simulations adopt the assumption of a uniform global magnetic field, which cannot reflect the magnetic field gradient along the arc length of the racetrack coil [19,41]. Refined coil segmentation is widely adopted to obtain the spatial magnetic-field distribution [42,43,44]. In this work, the coil is equally segmented with 5 mm intervals along the extension direction. Considering the symmetrical structure of the racetrack coil, the length (75 mm) on both sides of the central axis of the cross section is selected as one calculation module, as shown in Figure 3. We further decompose it into 15 sub-regions (5 mm), and the average magnetic field within each 5-mm segment is taken to represent the perpendicular field for the corresponding sub-region. A finite-element magnetic field calculation is performed to obtain the distribution of perpendicular magnetic flux density *B*_z_ inside each segment.

**Figure 4:**
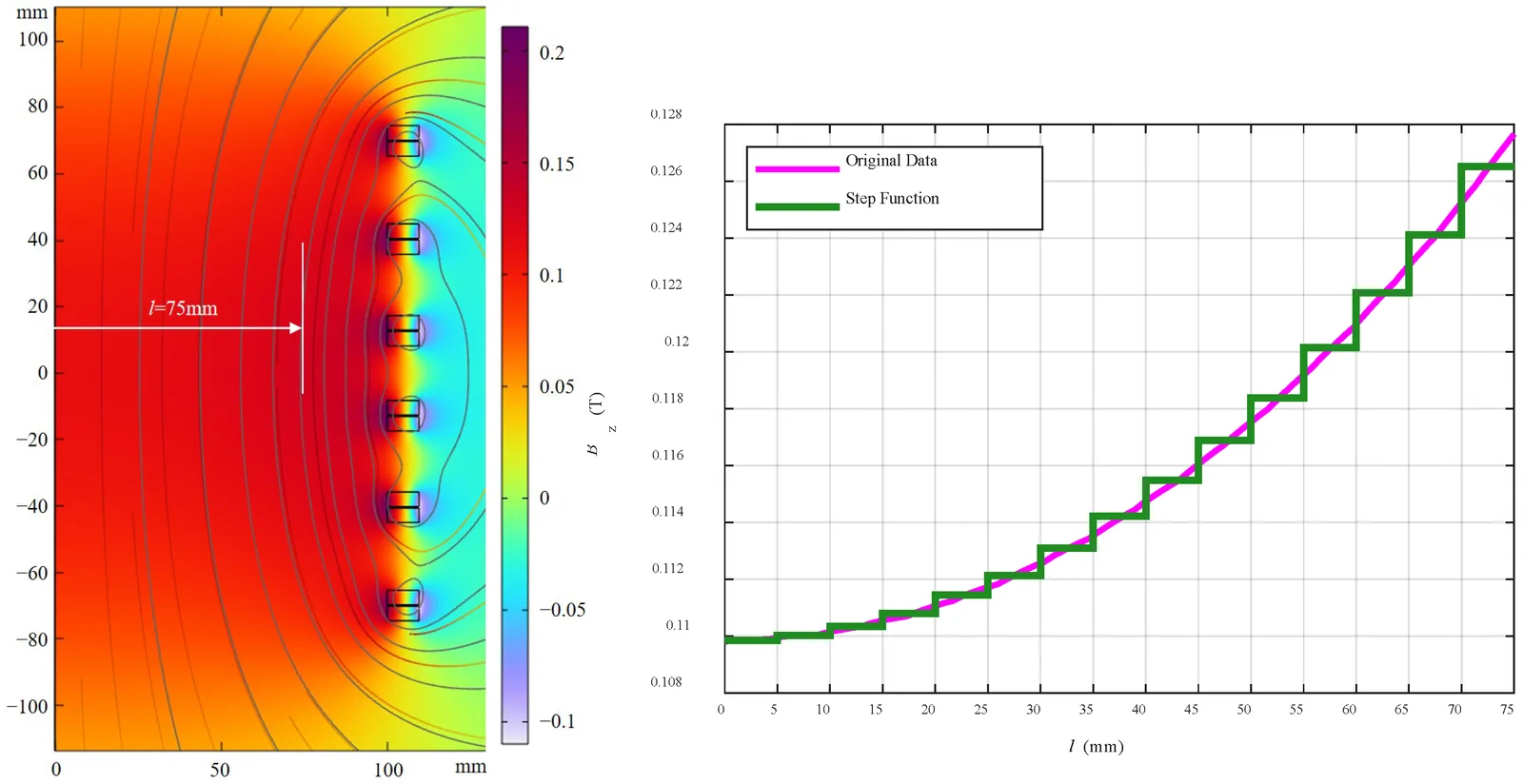
Simulation results of the external perpendicular field around the 15-segment RSFCL.

Significant spatial differences exist in the perpendicular magnetic field intensity inside the magnet [45,46,47]. Figure 4 presents the simulation results of the perpendicular magnetic field generated by the external solenoidal magnet. Along the conductor length of 0–75 mm, the perpendicular magnetic field *B*_z_ shows an overall monotonically increasing trend, with values ranging from 0.110 T to 0.128 T and a field difference of 0.018 T between the two ends of the coil. In addition, the magnetic field distribution along the arc length exhibits non-uniform and nonlinear characteristics: as the non-inductive coil gradually approaches the inductive solenoid, the magnetic field gradient increases progressively.

**Figure 5:**
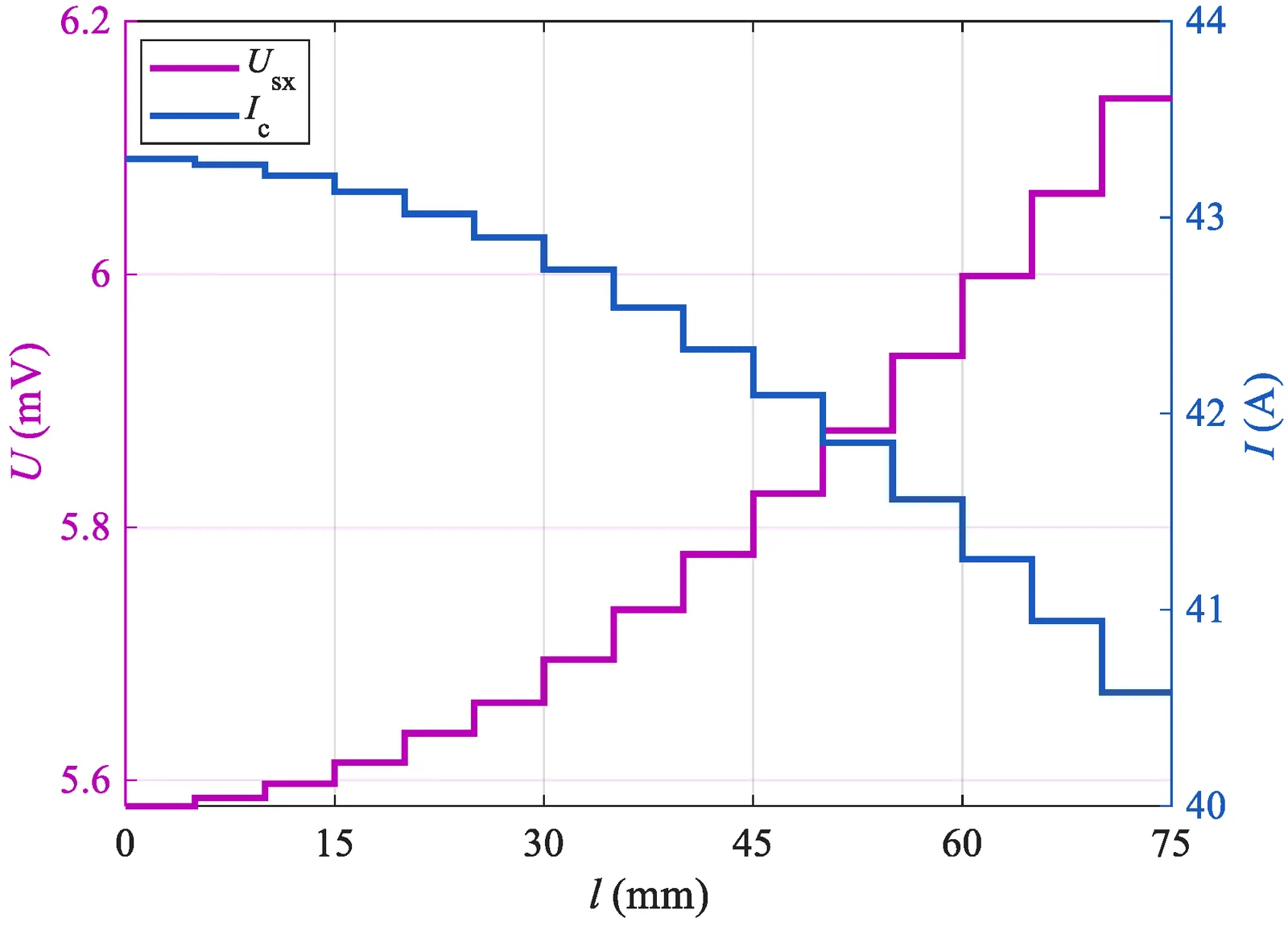
Terminal voltage and critical current distribution of each segment.

**Figure 6:**
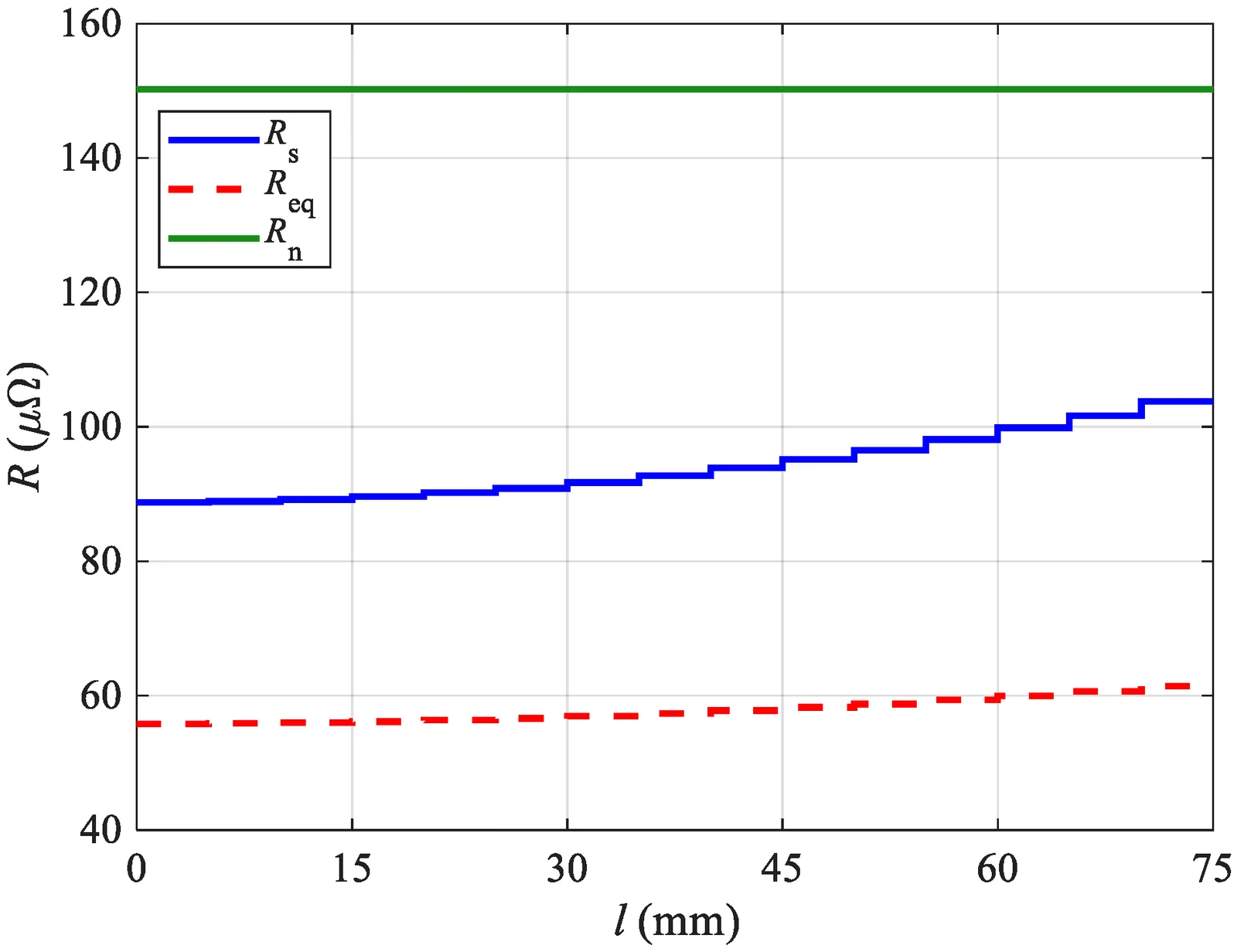
Resistance distribution of each segment.

Under a 100-A current charging condition for the quenching simulation, each coil segment shows differentiated stepwise degradation of current-carrying capability along the conductor length, driven by the gradient in the perpendicular background field. The transient critical current difference between the two ends reaches 2.72 A. Segments exposed to stronger magnetic fields exhibit lower critical currents. Under an identical current, these segments produce larger instantaneous equivalent resistances, accompanied by correspondingly higher voltage drops across each segment. Consequently, the whole coil exhibits a stepwise increasing distribution of segmented resistance and segmented voltage along the tape arc length, as illustrated in Figure 5 and Figure 6.

## 4. Fault-Current Limiting Characteristics Under Segmented Field

The electromagnetic-thermal coupled model proposed in this work is adopted to evaluate the fault-current limiting characteristics. All core simulation parameters required for Equations (1)–(12) are summarized in Table 3. It should be noted that the parameter values adopted in the model and formulas established in this paper are derived from experimental and simulation results reported in relevant literature. Deviations may occur in practical operation due to performance variations in superconducting materials and coils and in cryogenic refrigeration systems. In the actual design and development process of superconducting equipment, further testing and analysis are required to determine more accurate performance parameters for higher-precision model analysis.

**Figure 7:**
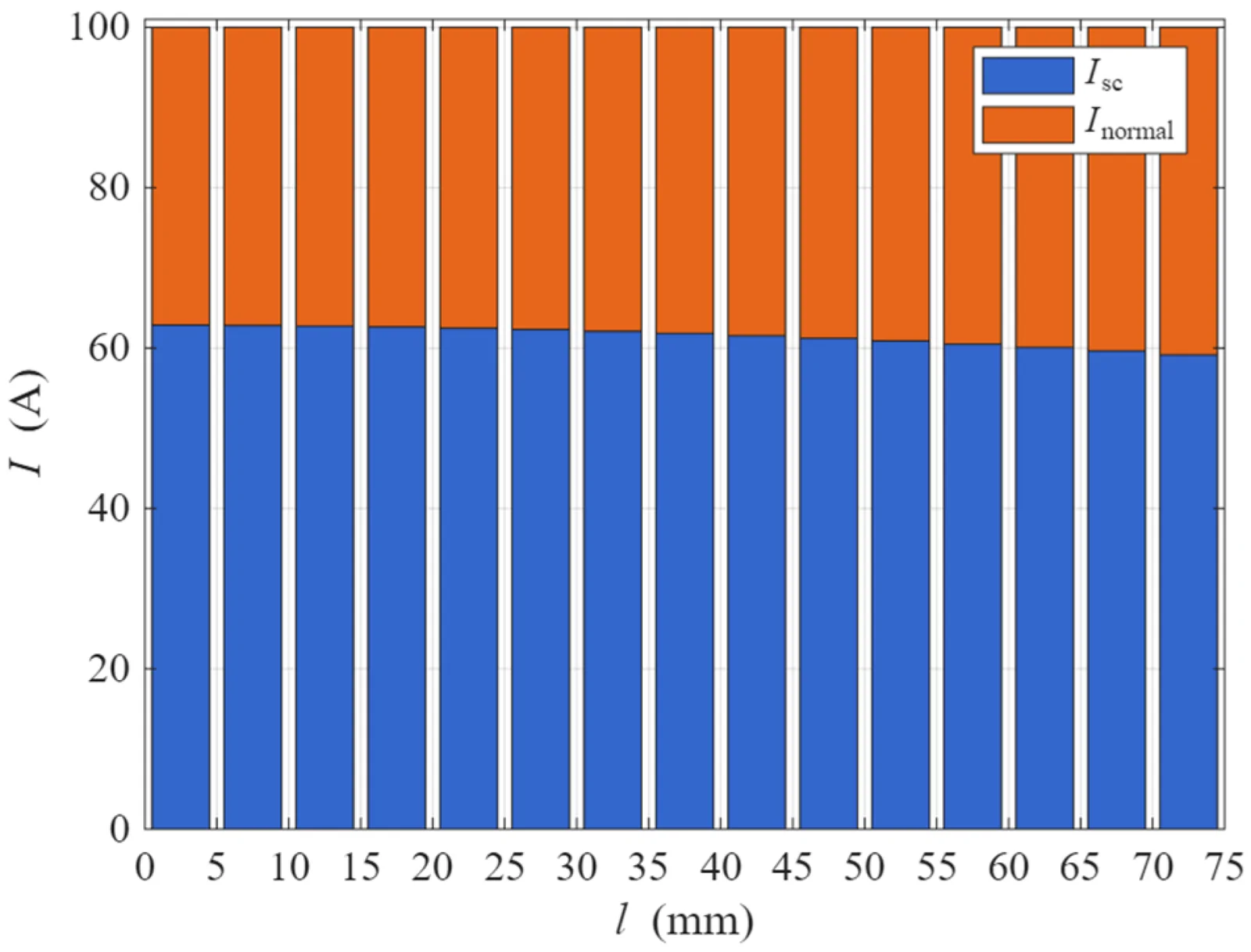
Current-sharing characteristics of the superconducting and metal layers in each segment.

Figure 7 presents the steady-state current-sharing distribution of the 15 segments under a prescribed transport current of *I*_s_ = 100 A. Due to the spatial discrepancy in the critical current *I*_c_ caused by different magnetic field intensities, the degree of current shunting varies at different positions. Regions with stronger magnetic fields correspond to lower critical currents, making it easier for the superconducting layers to enter nonlinear resistive states and diverting more current to the metal layers. The superconducting current difference between the two terminal segments reaches 3.72 A.

**Figure 8:**
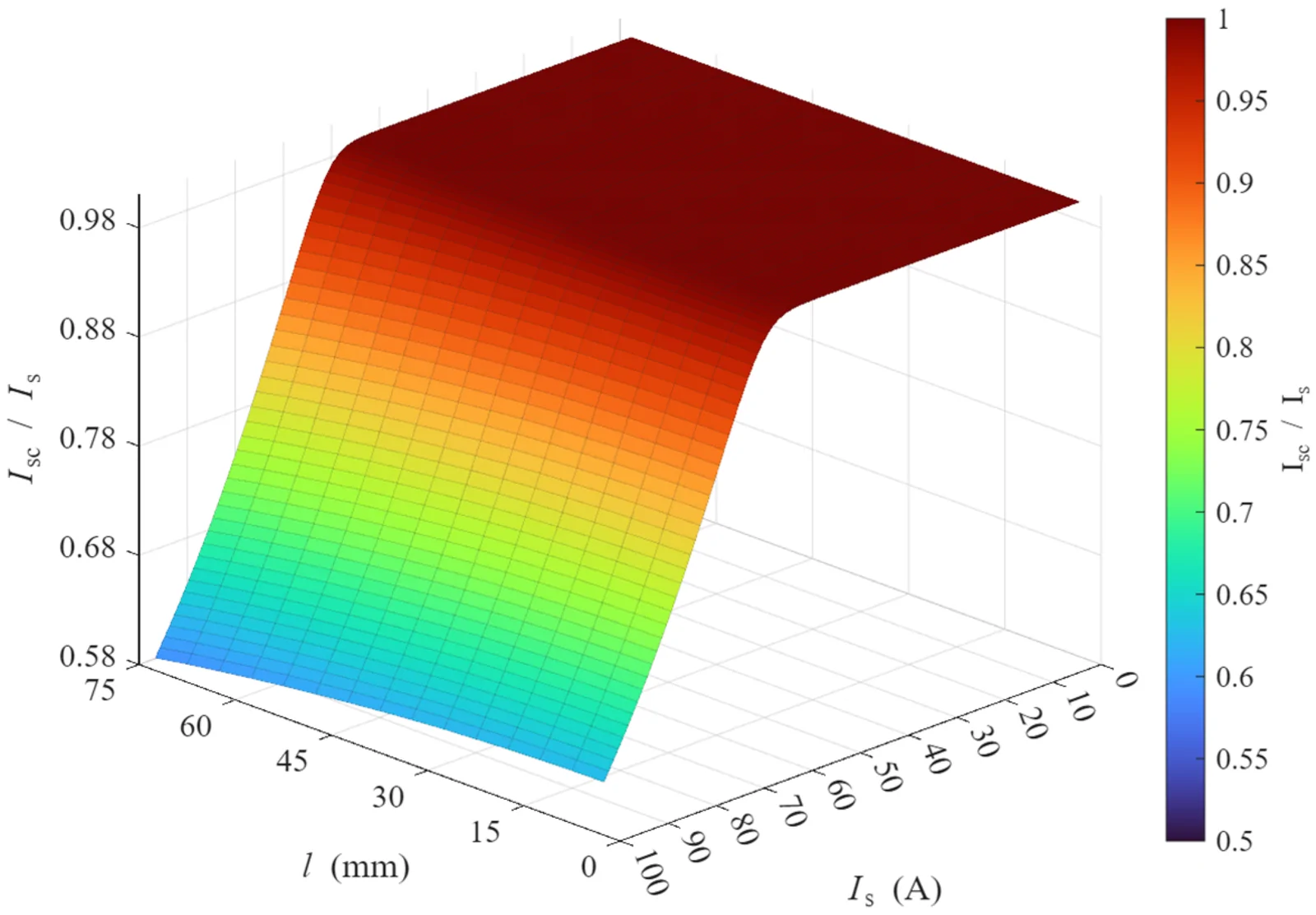
Variations in current-sharing proportions in the superconducting layer with operating current and arc length position.

Figure 8 further depicts the three-dimensional evolution relationship between the steady-state current proportion borne by the superconducting layers and position when the operating current *I*_s_ ranges from 0 A to 100 A. At a low operating current, the ratio *I*_sc_/*I*_s_ of each segment approaches 1, indicating that almost the whole current is carried by the superconducting layer. With the increase in the operating current, segments under high magnetic field witness their critical current drop below the operating current in advance, leading to rapid rise in quenching resistance and sharp decline in shunting proportion, forming stepwise shunting characteristics along the arc length.

### 4.1. Electromagnetic-Thermal Coupling Analysis Under Dynamic Fault-Current Excitation

**Figure 9:**
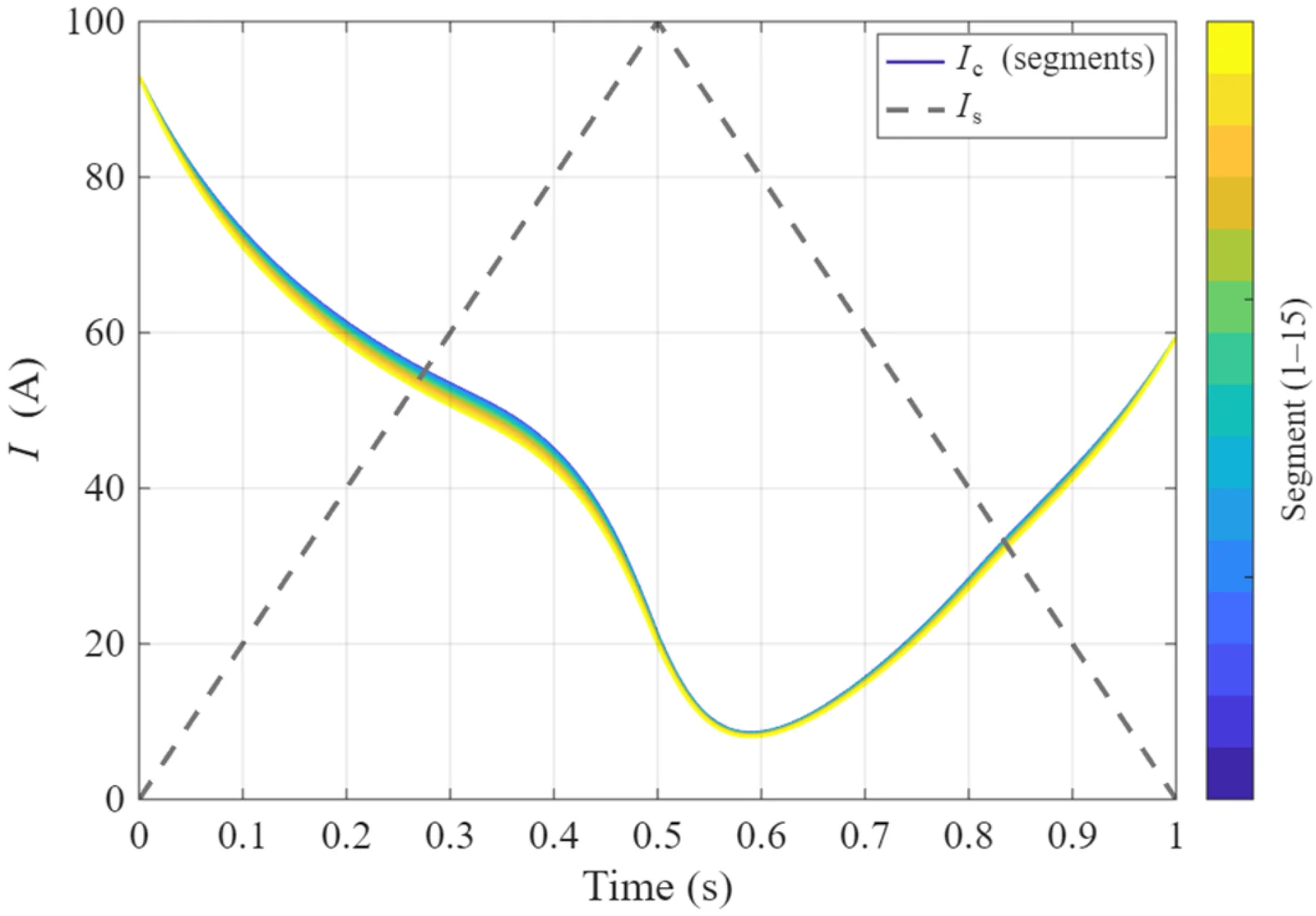
Dynamic variations in the critical current for each coil segment.

Modulating the waveform and amplitude of the injected current to simulate diverse operating conditions of fault-current limiters is a widely adopted simulation method [48,49,50]. The fault current is equivalent to the time-dependent operating current *I*_s_(*t*). Figure 9 demonstrates the dynamic variation in a fault-current limiter’s critical current when the prescribed time-dependent current waveform is applied.

During 0–0.5 s, the circuit current increases linearly, and the background magnetic field coupled by solenoidal magnet continuously and simultaneously strengthens. Combined with the performance attenuation induced by the Joule temperature rise, the critical current of all segments decreases monotonically. Affected by winding thermal inertia, the Joule heat accumulation lags behind the current variation, and the minimum critical current of the winding appears at *t* = 0.59 s. With the gradual decline in the circuit current during 0.5–1 s, the background magnetic field weakens synchronously, and the liquid nitrogen boiling continuously removes the accumulated heat of the winding, leading to a gradual recovery of the critical current in each segment.

**Figure 10:**
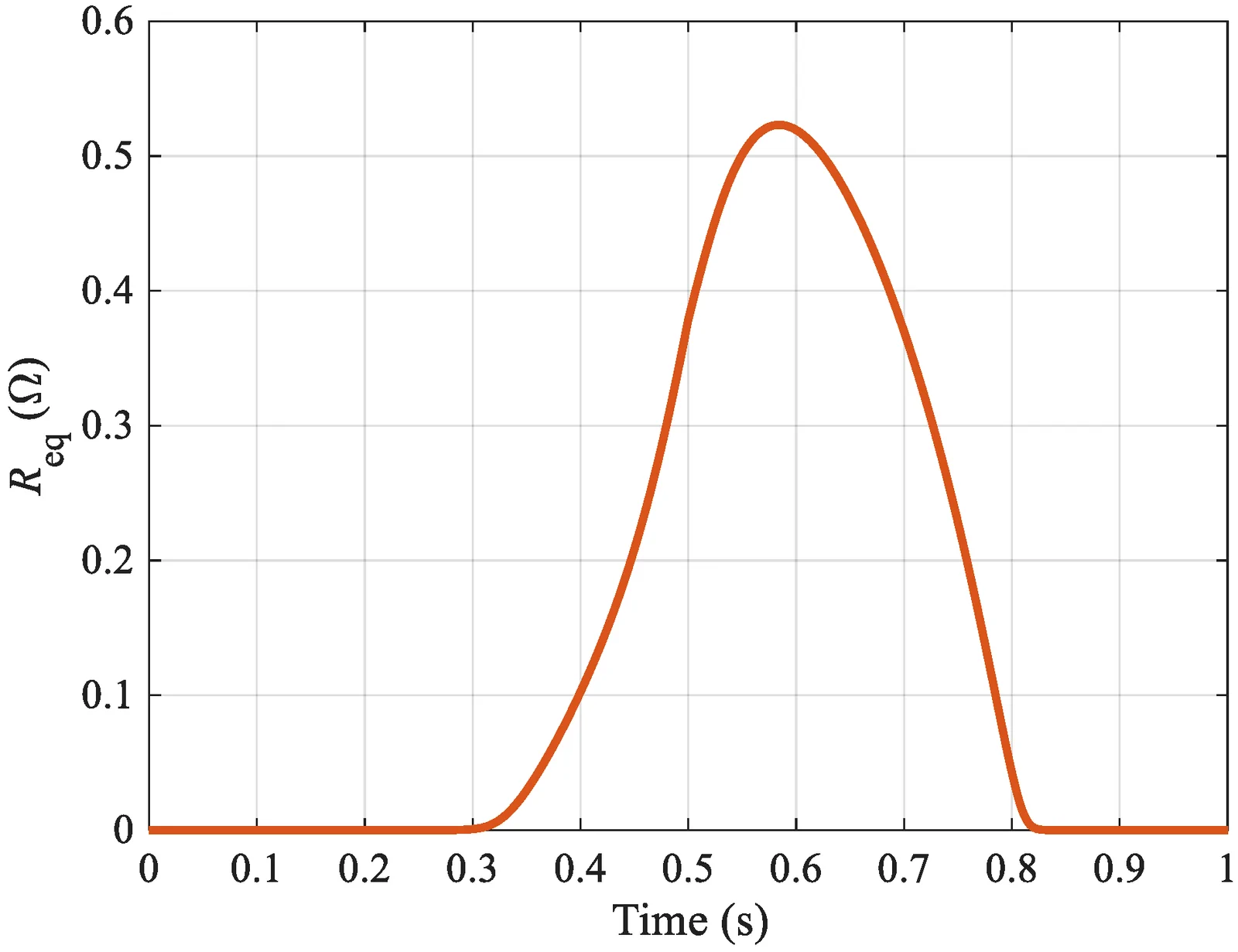
Dynamic variations in the equivalent resistance of the whole coil.

Starting from *t* = 0.27 s, when the critical current drops below the operating current, the equivalent resistance rises rapidly from approximately 5 × 10^−5^ Ω to 0.52 Ω within 0.3 s, as shown in Figure 10. Meanwhile, the voltage across the superconductor rises sharply, which significantly mitigates the impact of peak fault current on power devices and lines. As the operating current decreases, the superconducting equivalent resistance drops rapidly, and the winding fully recovers a zero-resistance superconducting state at *t* = 0.83 s.

**Figure 11:**
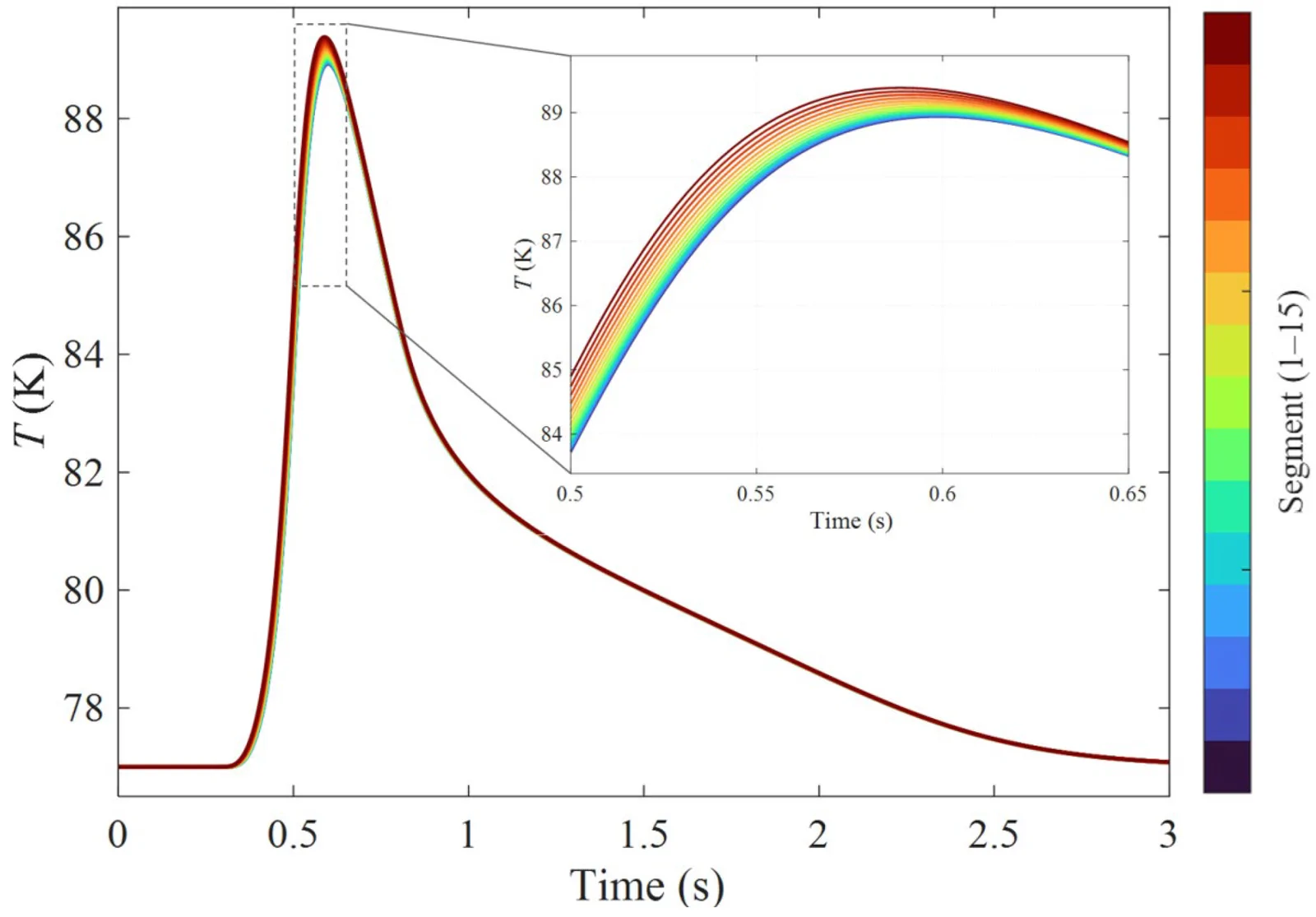
Dynamic temperature variation in the coil.

**Figure 12:**
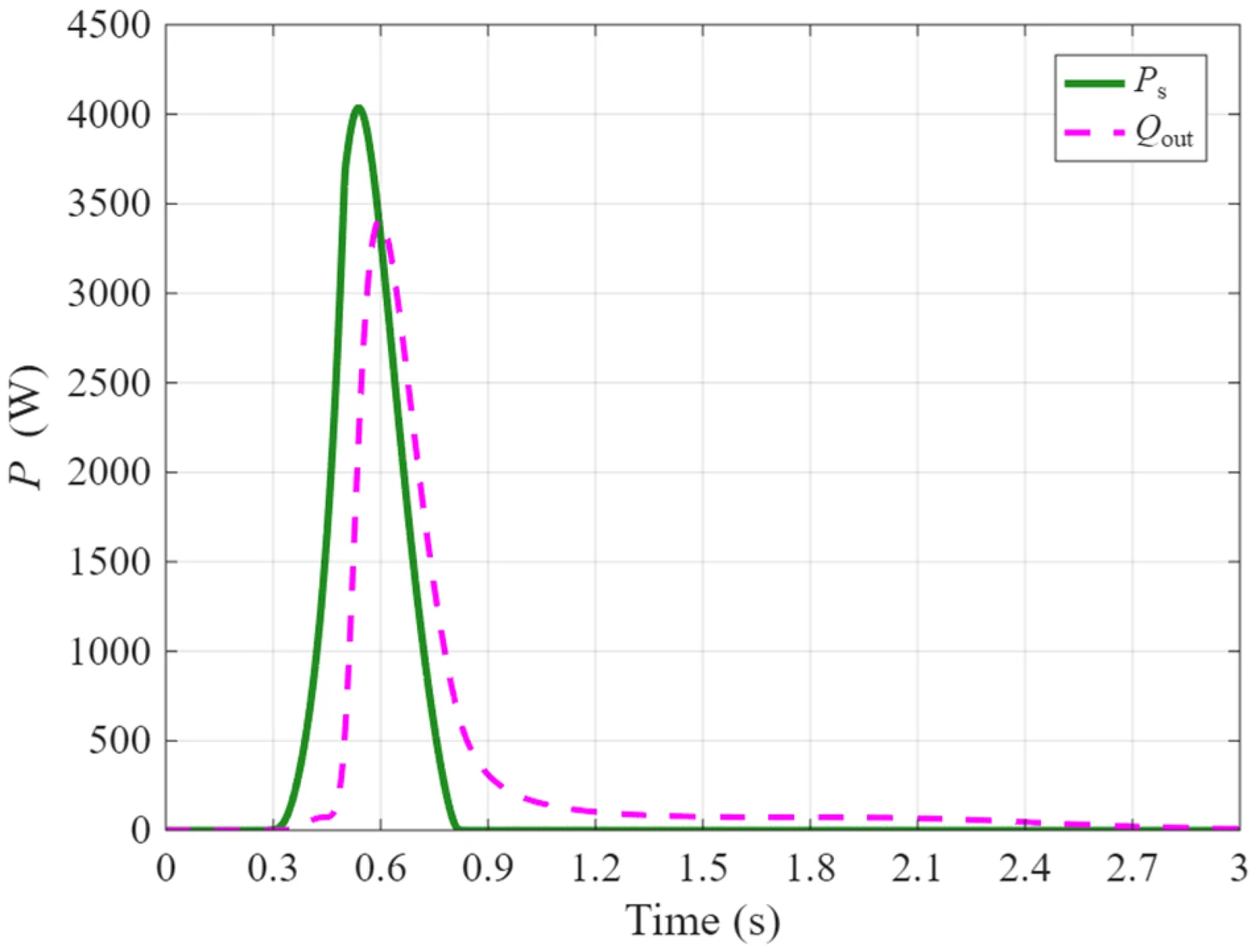
Dynamic power variation in the coil.

Figure 11 and Figure 12 present the segment-wise temperature evolution and global power response during the fault operation. From *t* = 0 to 0.27 s, the circuit current remains below the instantaneous critical current. Therefore, the equivalent resistance of the superconducting element approaches zero, and the temperature remains stable at 77 K.

During the fault-current-limiting interval from *t* = 0.3 to 0.6 s, the equivalent resistance of the superconducting element increases rapidly, leading to a sharp increase in power dissipation. The resulting Joule heating causes the conductor temperature to rise rapidly to a peak of approximately 89.4 K. At this instant, the temperature difference between the inner and outer winding segments is approximately 0.5 K, primarily driven by the spatial non-uniformity of the background magnetic field distribution. Specifically, segments subjected to stronger local fields feature lower critical currents and enter the resistive state earlier, leading to intensified local Joule dissipation. This local heating further degrades the critical current via the $I_{c}(T,B_{z})$ relationship, which in turn widens the temperature disparities between the different segments.

After *t* = 0.6 s, the fault current decreases below the critical current, and the equivalent resistance and Joule power dissipation decrease rapidly. Due to thermal inertia, the temperature decay exhibits a noticeable delay relative to the power variation. Under continuous liquid-nitrogen cooling, the overall winding temperature returns to 77 K within approximately 3 s.

**Figure 13:**
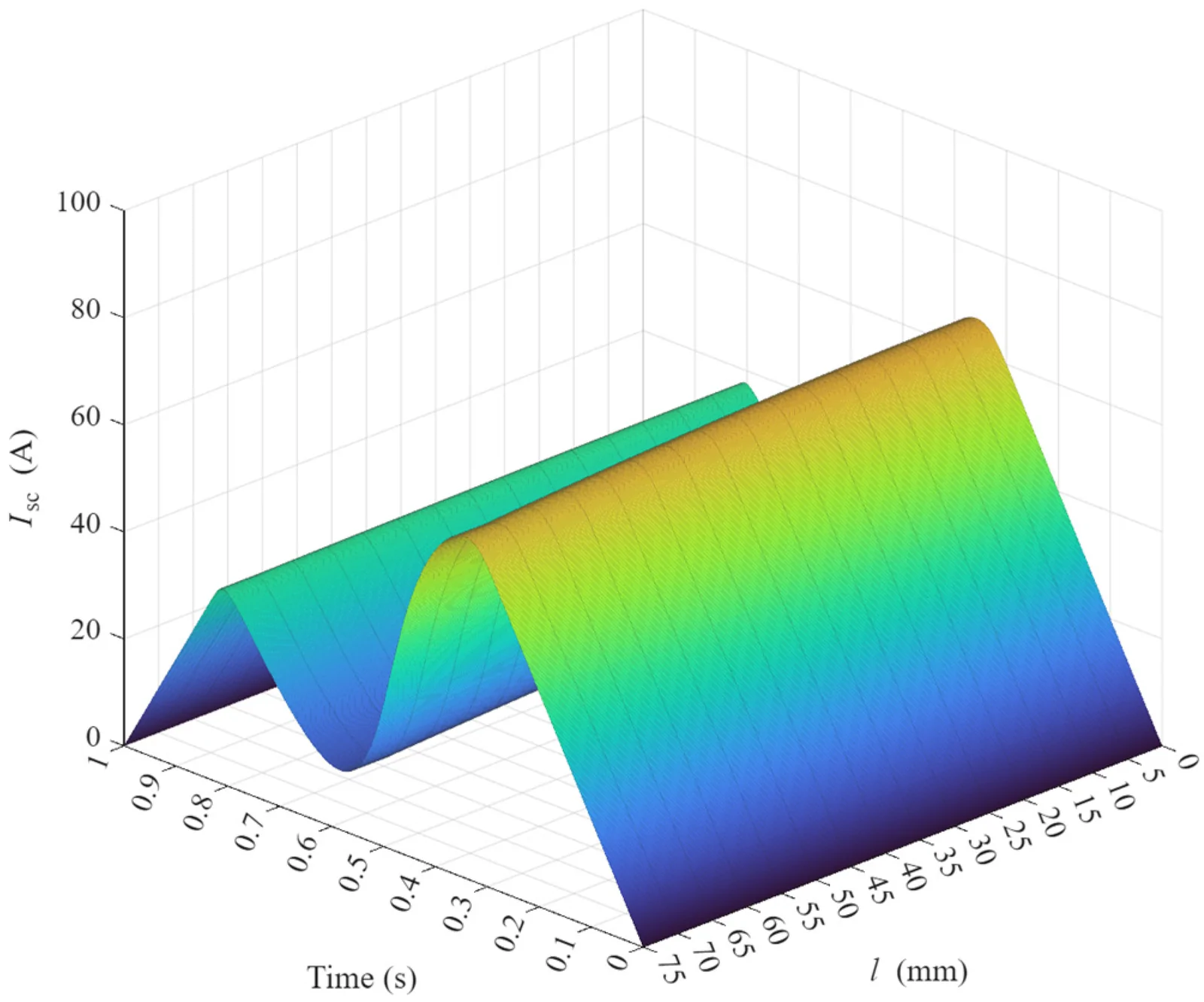
Dynamic variation in the superconducting current.

**Figure 14:**
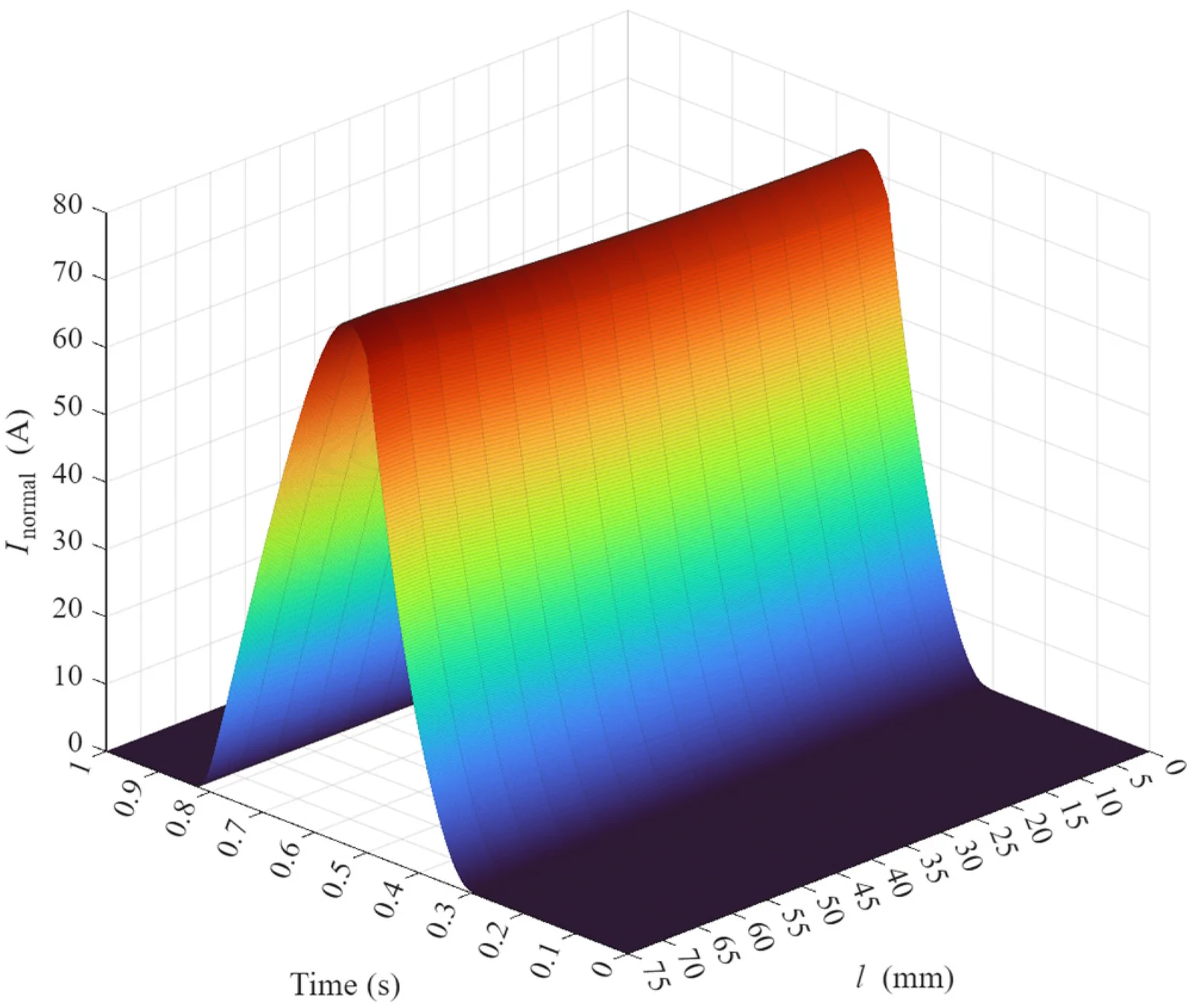
Dynamic variation in the sharing current in the metal layers.

Figure 13 and Figure 14 illustrate the evolution characteristics of currents in superconducting and the normal-conducting layers under dynamic current excitation. It can be observed that at a low current amplitude, *I*_sc_/*I*_s_ is close to 1, meaning the superconducting layer remains nearly lossless. As the operating current rises, the critical current continues to decrease under the combined impact of temperature rise and enhanced magnetic field. When the local current exceeds the critical current of the corresponding segment, the superconducting layer enters a nonlinear resistive state, and the current begins to divert from the superconducting layer to the metal layers, which results in a rapid drop in the current sharing proportion. For instance, the superconducting layers of all segments cover approximately 14.8% of the average operating current at *t* = 0.58 s. The maximum sharing current of metal layers reaches 71.61 A, but the superconducting current is only 12.48 A.

Leveraging the inherent fault self-recovery characteristic of the superconducting layer [9,51,52], as the fault current decreases continuously, the superconducting layer can gradually revert to its zero-resistance superconducting state once the local transport current drops below the recovery critical current. The shunt current in the metallic layers then flows back into the superconducting layer, and the proportion of superconducting current increases accordingly.

### 4.2. Comparison of Response Characteristics Under Actual Fault Conditions

**Figure 15:**
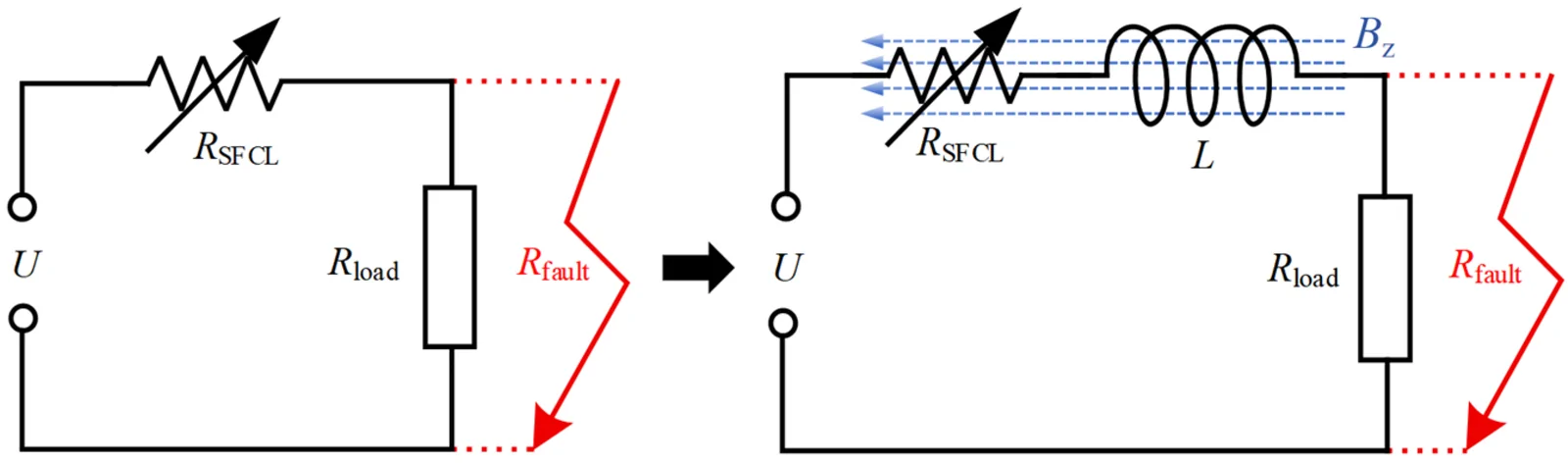
The equivalent circuit models in a conventional RSFCL and the improved SCLR system.

In this section, a DC short-circuit simulation model is established, with the constant-voltage-source short-circuit modeling framework proposed in [19] adopted as the simulation benchmark. This unified simulation framework allows for a quantitative evaluation of how the background magnetic field contributes to fault-current limitation. A metallic short-circuit fault is set to occur at *t* = 0.2 s and cleared at *t* = 0.4 s. The equivalent circuit of the proposed SCLR with embedded magnetic field excitation is compared with that of the conventional RSFCL, as illustrated in Figure 15.

**Figure 16:**
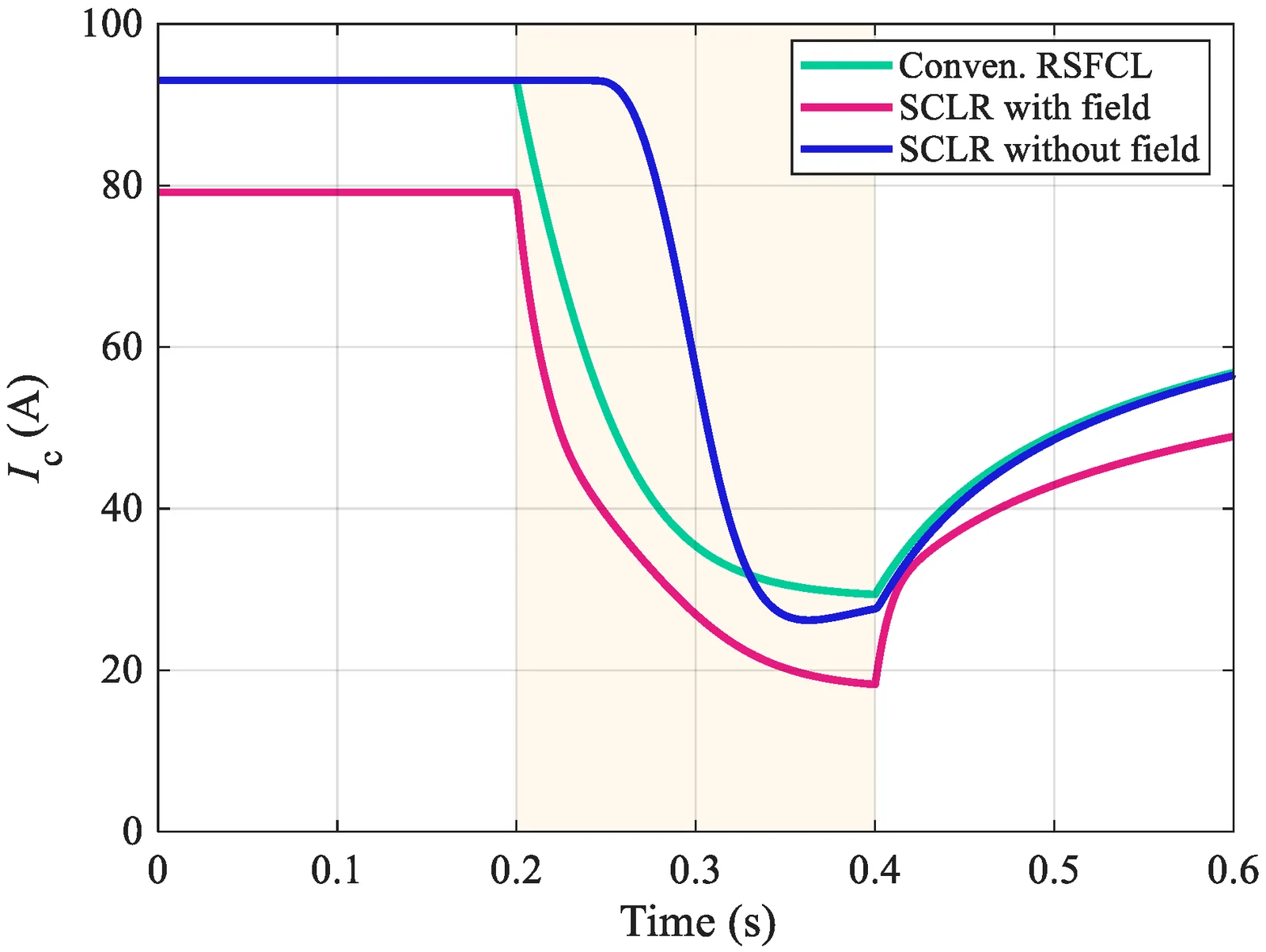
Critical current degradation comparison.

**Figure 17:**
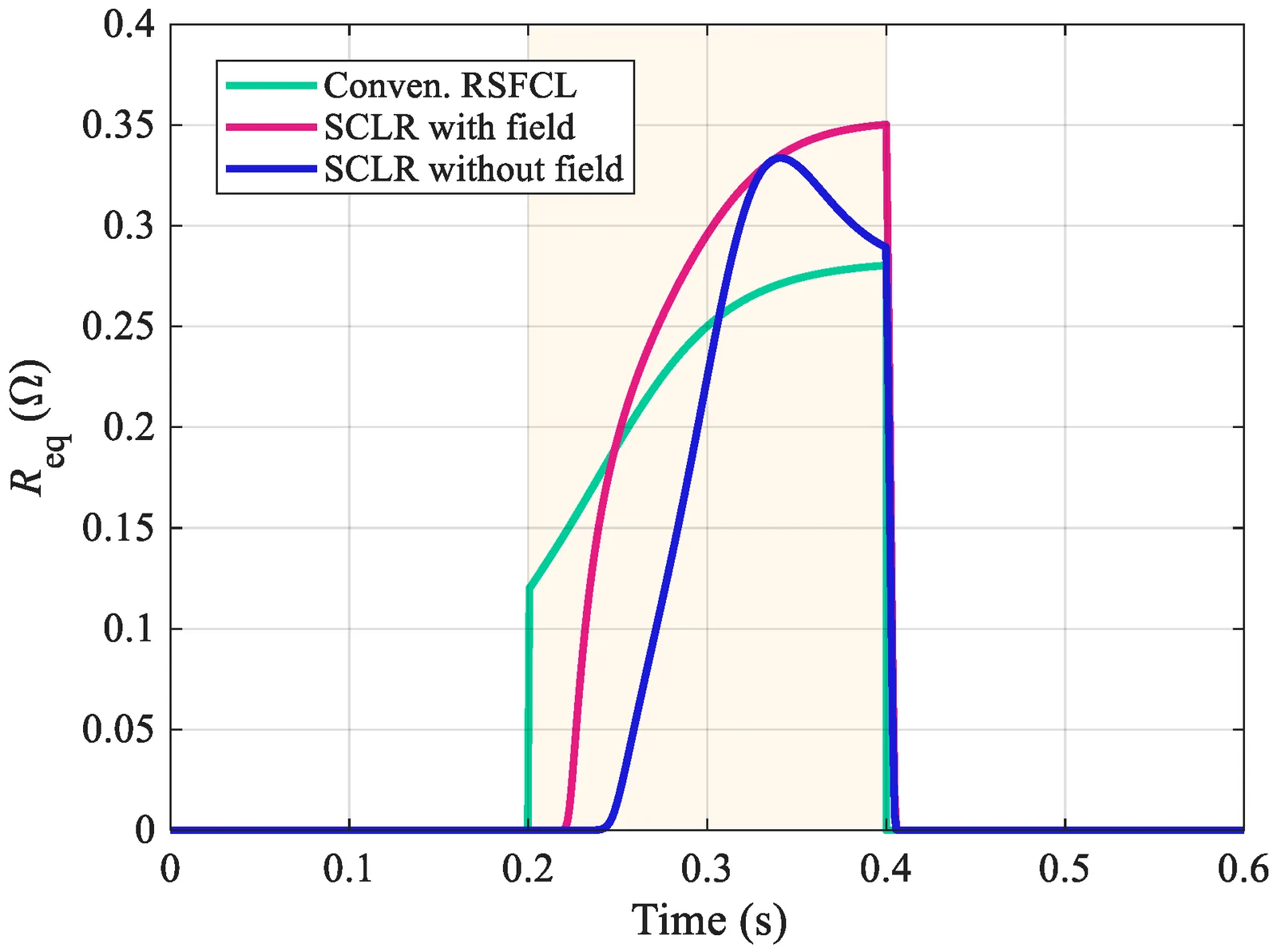
Equivalent current-limiting resistance comparison.

Figure 16 and Figure 17 compare the temporal evolution of current and resistance for three types of fault-current limiter configurations throughout the fault period, with the fault initiated at *t* = 0.2 s.

The conventional RSFCL, lacking series inductance, exhibits a sharp rise in fault current that triggers rapid Joule-heating-induced quenching, establishing its equivalent resistance almost instantaneously. In contrast, the series inductance in the proposed SCLR without background field excitation suppresses the rising rate of fault current, delaying both critical current degradation and resistance buildup by approximately 40 ms. For the SCLR with background field excitation, a pre-applied magnetic field reduces the initial critical current from 93 A to approximately 79.1 A, driving the tape into a resistive state immediately upon fault onset. Compared to the field-free SCLR, this configuration cuts response time by roughly 50%, induces more significant critical current degradation, and lowers the critical current to a minimum of 18.2 A during the fault.

Since the conventional RSFCL relies exclusively on thermal effects to initiate quenching, the growth of its equivalent resistance slows in the mid-to-late fault stages. While the quenching initiation is delayed in the field-free SCLR, sustained heat accumulation promotes quenched region expansion as the fault progresses, causing its equivalent resistance to increase continuously and reach a peak of approximately 0.33 Ω in the mid-to-late fault period. Throughout the fault interval, the field-assisted SCLR maintains a consistently lower critical current and achieves the highest equivalent resistance among the three configurations in the mid-to-late fault stages, peaking at 0.35 Ω. These features highlight its exceptional current-limiting performance.

**Figure 18:**
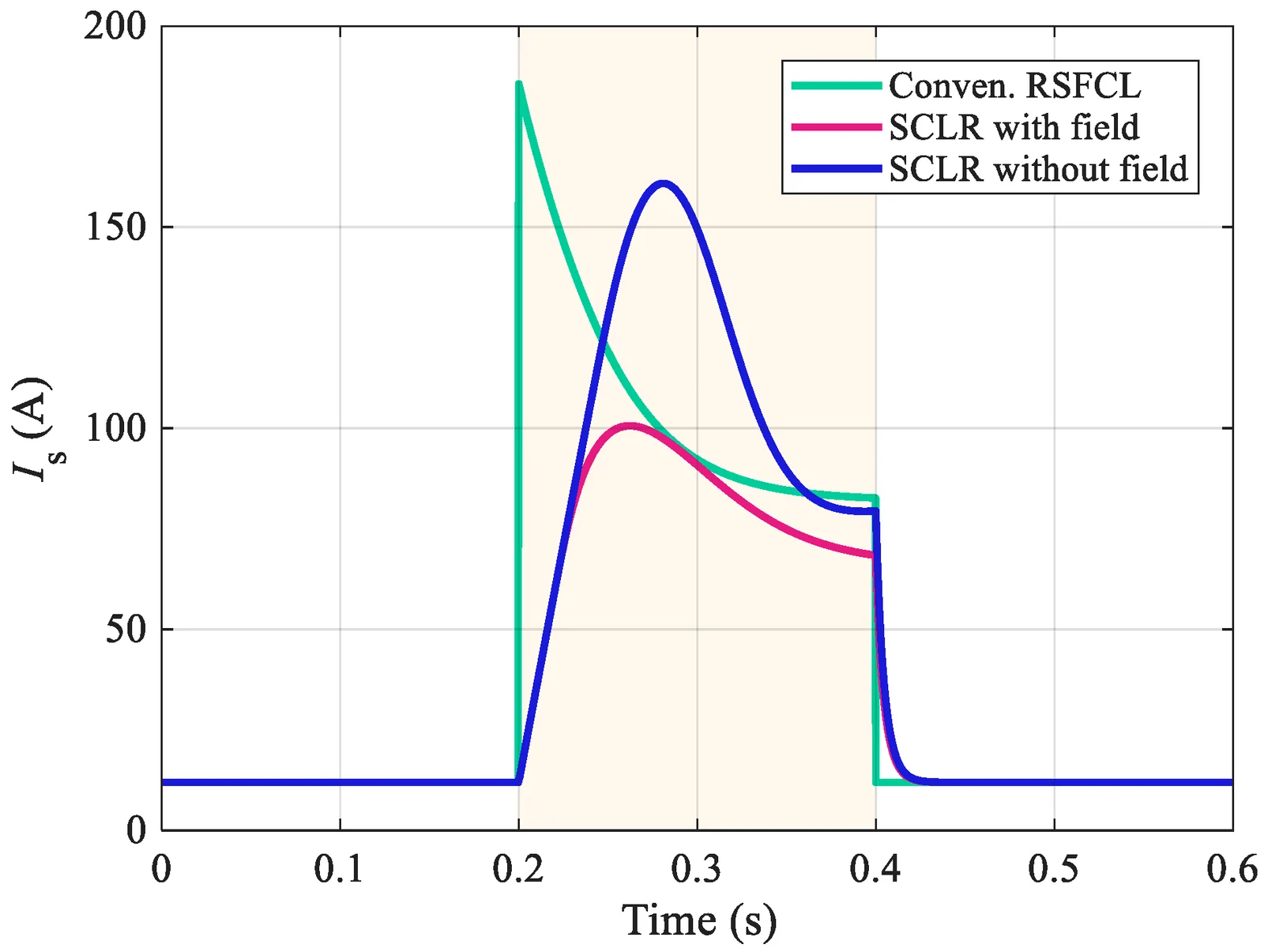
Line current response comparison.

Figure 18 illustrates the dynamic evolution of the fault current across the three current-limiting configurations. For the conventional RSFCL, the fault current surges instantaneously to a peak of approximately 185 A at fault onset, followed by a monotonic decay. In contrast, the series inductance in the field-free SCLR mitigates the rate of current rise, resulting in a more gradual increase that peaks at 160 A at *t* = 0.28 s. The field-assisted SCLR reaches a peak fault current of approximately 100 A at *t* = 0.26 s, marking the lowest peak among the three configurations.

In terms of current-limiting performance, the peak current of the field-assisted SCLR is 37.5% lower than that of the field-free SCLR and 45.9% lower than that of the conventional RSFCL. Throughout the entire fault duration (0.2–0.4 s), the fault current of the field-assisted SCLR remains consistently below the levels observed in the other two configurations. These simulation results suggest that the background magnetic field significantly enhances the fault-current suppression capability of the SCLR.

**Figure 19:**
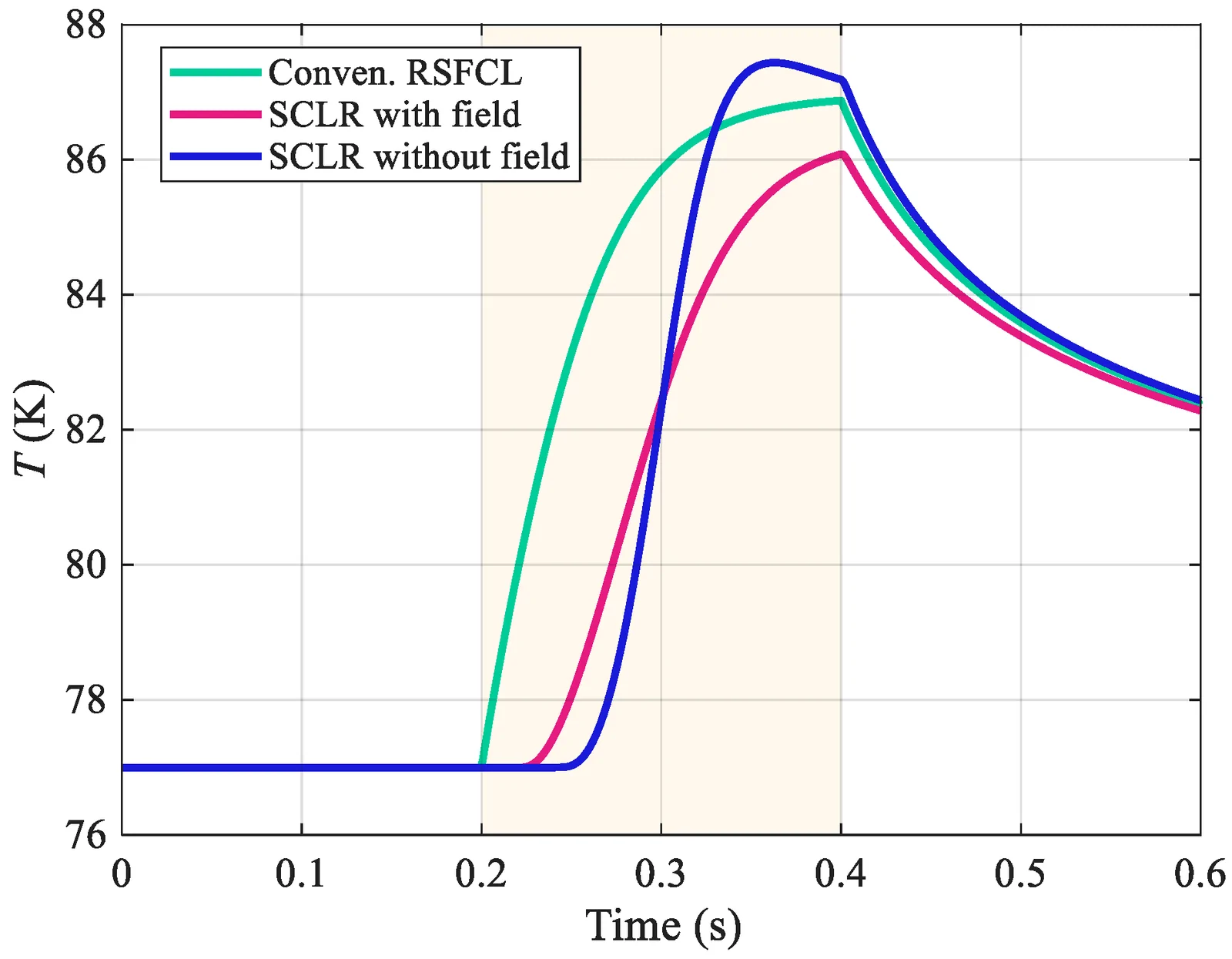
Tape temperature comparison.

Figure 19 compares the temporal evolution of superconducting temperature across the three current-limiting configurations. At fault inception, the conventional RSFCL responds instantaneously, triggering immediate Joule heating and a corresponding temperature rise in the superconducting winding. Instantaneous power dissipation reaches approximately 4100 W at fault onset, and the winding temperature increases steadily as the fault persists. For the field-free SCLR, the series inductance suppresses the rate of fault-current rise while extending the duration of elevated power dissipation. Consequently, power dissipation peaks at approximately 5100 W during the fault, driving the maximum winding temperature up to 87.4 K.

In contrast, the integrated background field allows the proposed field-assisted SCLR to enter the quenching state much more rapidly. This results in a more gradual and lower-magnitude winding temperature rise, with peak power dissipation limited to approximately 2500 W. This configuration effectively mitigates the short-duration thermal shock to the superconducting tape and reduces total heat generation within the winding. Compared to the field-free SCLR, the maximum winding temperature rise is reduced by approximately 1.4 K.

### 4.3. Parametric Sensitivity Analysis of Power Supply Voltage

**Figure 20:**
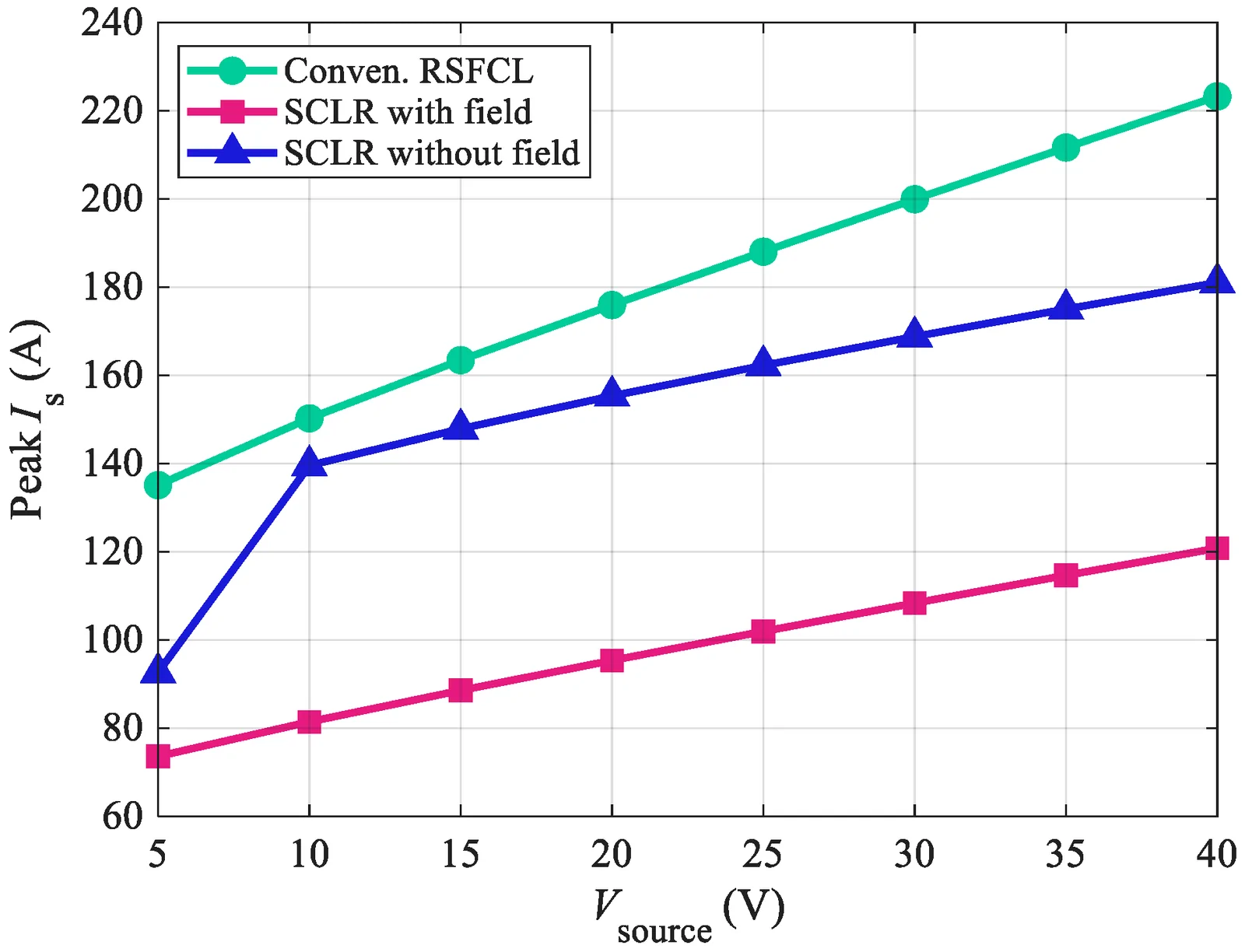
Comparison of maximum fault current under different source voltages.

**Figure 21:**
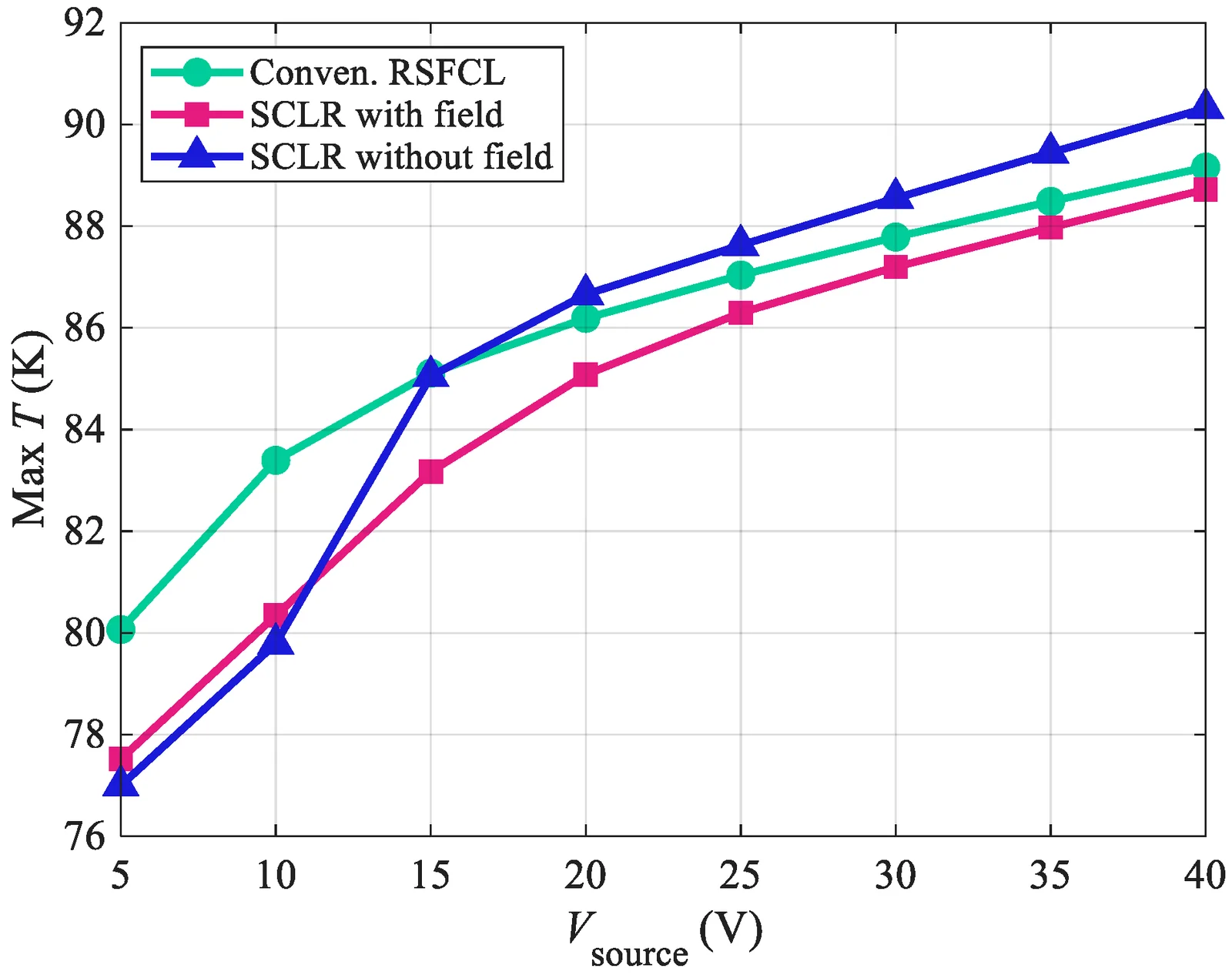
Comparison of maximum fault temperature under different source voltages.

For the parameter sensitivity analysis, the DC source voltage is set to increase from 5 V to 40 V. Figure 20 depicts the variation in the peak fault current for different source voltages, while Figure 21 further compares the peak winding temperature characteristics of the three current-limiting configurations.

The conventional RSFCL undergoes an immediate resistive transition at fault inception due to the abrupt surge in short-circuit current. Across the 5–40 V range, both its current-limiting capability and thermal response exhibit monotonic variations. In contrast, the operating characteristics of the SCLR are more sensitive to fault voltage due to its integrated series inductance.

In the low-voltage region below 5 V, the field-free SCLR is constrained by its series inductance. Although the fault current rises to nearly 90 A, it remains below the critical current threshold, failing to trigger noticeable resistive transition and temperature rise. By comparison, the field-assisted SCLR can rapidly initiate resistive transition within this low-voltage range, effectively limiting the fault current to approximately 73 A, though its maximum winding temperature is slightly higher than that of the field-free SCLR.

When the source voltage exceeds 10 V, the winding of the field-free SCLR rapidly enters the resistive state at a relatively high current level, leading to concentrated Joule heating and a sharp temperature increase. Its maximum temperature rises to approximately 85.1 K at 15 V. The performance advantage of the field-assisted SCLR becomes increasingly pronounced above 10 V, where it maintains both the lowest peak current and the lowest maximum temperature. At 40 V, its maximum fault current is approximately 120 A, representing reductions of 46.19% and 33.71% compared with the conventional RSFCL and the field-free SCLR, respectively.

These simulation results demonstrate that the introduction of a background field effectively mitigates the inherent quenching delay of the proposed field-assisted SCLR, prevents localized heat accumulation under high-voltage fault conditions and significantly enhances the thermal stability across a wide range of fault severities.

## 5. Conclusions

A novel racetrack-type SCLR topology integrated with a solenoid-biased background magnetic field has been investigated in this paper. The racetrack current-limiting coil is embedded within a solenoidal magnet. This configuration fundamentally mitigates the response delay inherent in conventional temperature-triggered SFCL topologies. It enables rapid superconducting quench upon fault occurrence while suppressing fault current simultaneously. Compared with the SFCL without a built-in bias magnetic field, the proposed device cuts the response time by 50% and cuts the peak fault current by 37.5%. Moreover, the maximum temperature rise of the winding is 1.4 K lower than that of the conventional RSFCL.

A fully coupled electromagnetic-thermal time-domain simulation framework has been developed through integration with a segmental field-dependent critical current model. This framework enables quantitative evaluation of the transient electrical and thermal responses triggered by spatially non-uniform perpendicular magnetic fields. Our developed multi-field coupled simulation model successfully reproduces core characteristics of the magnetic-field-accelerated quenching process, thereby providing a feasible numerical tool for structural parameter optimization of magnetically controlled SFCLs and SCLRs.

## Figures and Tables

**Table 1:** Main parameters of the racetrack-type coil.

Parameter	Value
Turn number	25
Single-turn width	4.4 mm
Coil width	25.6 mm
Straight segment length	150 mm
Coil thickness	11.35 mm
Total tape length	17.56 m

**Table 2:** Main parameters of the solenoid magnet.

Parameter	Value
Coil number	2
Axial gap	0.4 mm
Height	9.6 mm
Assembly number	6
Inter-axial gap	A1-A2, B1-B2: 20 mm A2-A3, B2-B3: 18 mm A3-B3: 16 mm
Height	149.6 mm
Inner radius	100 mm
Outer radius	109.6 mm

**Table 3:** Main simulation parameters for the proposed SCLR.

Parameter	Value	Unit	Description
*n*	25	-	Power-law index
*E*_c_	$1\;\times \;10^{-4}$	V/m	Critical field strength
*T*_0_	77	K	Initial operating temperature
$I_{c(T_{0})}$	93	A	Initial critical current
*T*_c_	93	K	Critical temperature
*α*	0.65	-	Anisotropic factor
*β*	1.2	-	Temperature exponent
*B*_1_	0.049	T	Anisotropic factor
*m*	0.1296	kg	Mass of racetrack coil
*l_t_*	17.56	m	Total tape length of racetrack coil
*L*	10	mH	Inductance of the smoothing inductor
*S_Ag_*	$1.5\;\times \;10^{-8}$	m^2^	Cross-sectional area of silver layer
*S_Cu_*	$5\;\times \;10^{-8}$	m^2^	Cross-sectional area of copper layer
*S_SS_*	$8\;\times \;10^{-7}$	m^2^	Cross-sectional area of stainless steel layer
*K_Ag_*	0.0065	1/°C	Silver resistivity temperature coefficient
*K_Cu_*	0.0065	1/°C	Copper resistivity temperature coefficient
*K_SS_*	0.087	1/°C	Stainless steel resistivity temperature coefficient
ρ *_Ag_*	$0.27\;\times \;10^{-8}$	Ω·m	Resistivity of silver at 77 K
ρ *_Cu_*	$0.19\;\times \;10^{-8}$	Ω·m	Resistivity of copper at 77 K
ρ *_SS_*	$56.8\;\times \;10^{-8}$	Ω·m	Resistivity of stainless steel at 77 K

## Data Availability

The raw data supporting the conclusions of this article will be made available by the authors on request.
